# A Large Substernal Goiter that Extended to Both Sides of the Thorax

**DOI:** 10.1155/2018/6107982

**Published:** 2018-11-05

**Authors:** Hirotaka Nakayama, Motohiko Goda, Kaori Kohagura, Nobuyasu Suganuma, Hiroyuki Iwasaki, Haruhiko Yamazaki, Soji Toda, Katsuhiko Masudo, Yasushi Rino, Munetaka Masuda

**Affiliations:** ^1^Department of Surgery, Yokohama City University, 3-9 Fukuura, Kanazawa-ku, Yokohama, Kanagawa 236-0004, Japan; ^2^Department of Breast and Endocrine Surgery, Kanagawa Cancer Center, 241-8515, 2-3-2, Nakao, Asahi-ku, Yokohama, Kanagawa, Japan; ^3^Department of Surgery, Yokohama Minami Kyousai Hospital, 236-0037, 1-21-1, Mutsuurahigashi, Kanazawa-ku, Yokohama, Kanagawa, Japan

## Abstract

Most substernal goiters can be managed through the transcervical approach, but a sternotomy is required in some cases. This report is about a large substernal goiter, which was resected via a transcervical and full sternotomy approach. The patient was a 57-year-old female, who visited our hospital for surgical treatment for a large substernal goiter. Computed tomography of the neck and chest revealed that the substernal goiter extended to both sides of the thorax and had compressed the trachea. We performed total thyroidectomy safely via a transcervical and full sternotomy approach. No postoperative complications occurred, except transient hypocalcemia. A histopathological examination did not reveal any malignancy, and the lesion was diagnosed as an adenomatous goiter. Most substernal goiters can be managed through the transcervical approach, but a full sternotomy is required when a substernal goiter extends to both sides of the thorax and/or has a larger diameter than the thoracic inlet or airway constriction is revealed. A full sternotomy provides excellent exposure and can help reduce the risk of complications, such as recurrent laryngeal nerve palsy and injuries to major blood vessels.

## 1. Introduction

Substernal goiters are occasionally encountered in patients with thyroid disease. The incidence of substernal goiters among patients with thyroid goiters is reported to range from 5.1 to 15.7% [1, 2]. There are various reported definitions of the condition, and the most commonly accepted definition is as follows: when >50% of the volume of a goiter extends below the thoracic inlet [3, 4]. Most substernal goiters are resectable via cervical manipulation alone, but sternotomy is required in a few cases. We report a case involving a large substernal goiter, which was safely resected via a transcervical and full sternotomy approach.

## 2. Case Presentation

**Figure 1:**
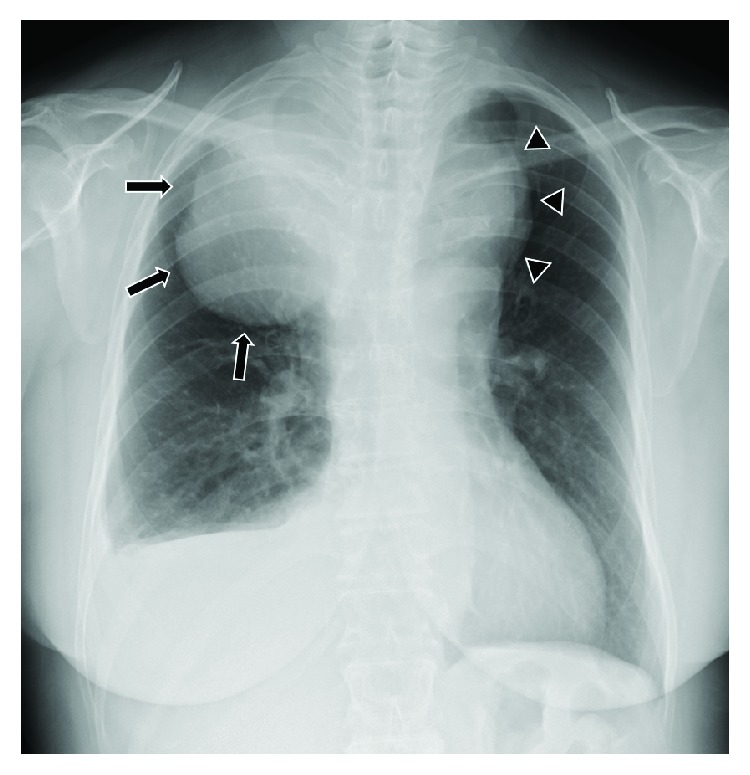
Chest X-ray findings. The chest X-ray showed a tumor shadow (arrow) in the upper-middle field of the right lung with pleural effusion and a tumor shadow (arrowhead) in the upper field of the left lung.

A 57-year-old female visited a respiratory internal physician due to suspected lung cancer (based on a mass screening chest X-ray examination). She did not have any symptoms. The chest X-ray showed a tumor shadow in the upper-middle field of the right lung with pleural effusion and a tumor shadow in the upper field of the left lung (Figure 1). Computed tomography (CT) of the neck and chest revealed that the tumor shadows had been caused by a substernal goiter connected to the thyroid gland in the neck. According to the patient, she had been diagnosed with a goiter about 23 years ago, and it was followed up, but the follow-up process had been discontinued several times. After about 20 years, she visited our hospital for surgical treatment.

In a physical examination, the palpable thyroid gland was found to be diffusely swollen and soft and exhibited poor mobility. The lower pole of the thyroid was not palpable.

A blood examination revealed normal thyroid function, a thyroglobulin level of 352 ng/ml, and negativity for the thyroglobulin antibody.

Ultrasound showed that the cervical thyroid gland was diffusely enlarged and exhibited multiple regions of cystic degeneration, but no obvious malignant findings were observed.

**Figure 2:**
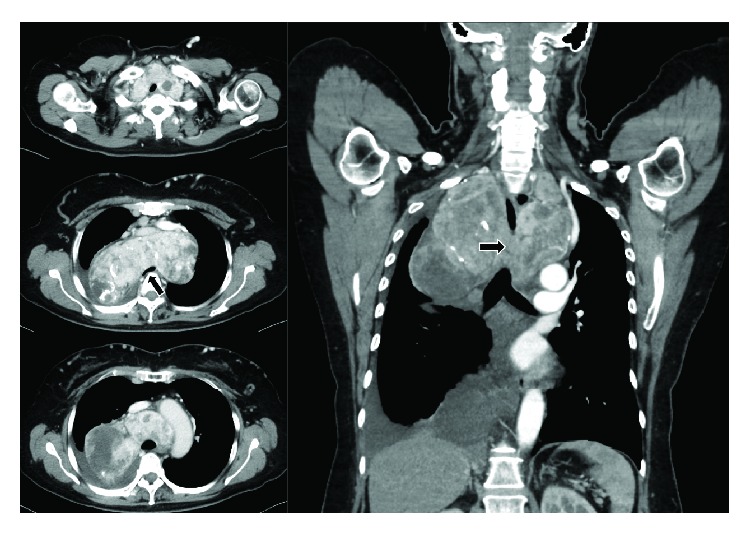
CT findings. CT of the neck and chest showed diffuse swelling of the thyroid gland and a substernal goiter, which extended to both sides of the thorax. The goiter extended to the bifurcation of the trachea on the dorsal side of the superior vena cava, the innominate vein, the aortic arch, and the ventral side of the trachea. It compressed the trachea in the mediastinum, and the lumen of the trachea measured 6 mm in diameter at its narrowest point (arrow). Pleural effusion was also seen in the right thorax.

CT of the neck and chest (Figure 2) showed the diffusely swollen thyroid gland and a substernal goiter, which extended to both sides of the thorax. Specifically, it extended to the bifurcation of the trachea on the dorsal side of the superior vena cava, the innominate vein, the aortic arch, and the ventral side of the trachea. The width of the goiter at the mediastinum was 145 mm (length: 80 mm, thickness: 80 mm). The right side of the substernal goiter was bigger than its left side. The interior of the lesion was heterogeneous, and calcification was seen in part of it. The goiter had compressed the trachea in the mediastinum, and the lumen of the trachea measured 6 mm in diameter at its narrowest point. Pleural effusion was noted in the right thorax. We performed 18F-fluorodeoxy glucose positron emission tomography to determine the malignancy of the substernal goiter, but no radiotracer accumulation was observed.

We also conducted a pathological examination. Fine-needle aspiration cytology of the cervical thyroid gland resulted in the lesion being classified as of “indeterminate significance,” and a pathological examination of a needle biopsy sample from the same site led to the lesion being diagnosed as a follicular neoplasm. Fine-needle aspiration cytology of the right pleural effusion demonstrated that it was benign.

**Figure 3:**
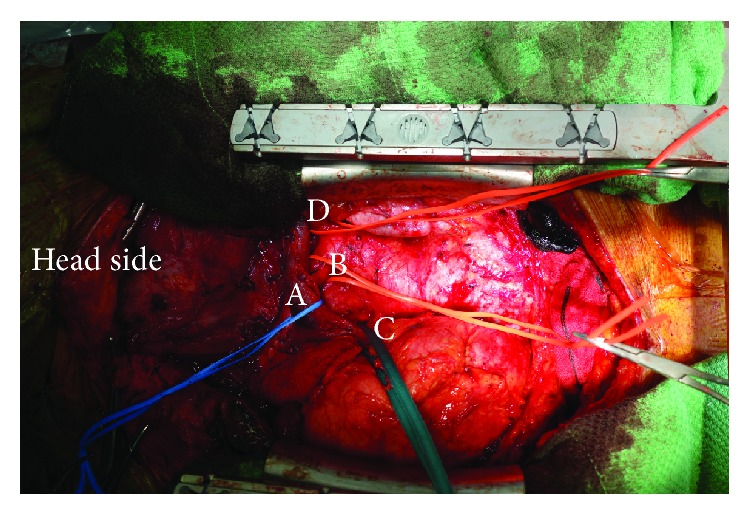
Surgical findings. As a preparation for the resection of the substernal goiter, the major blood vessels, such as the innominate vein (A), brachiocephalic trunk (B), vena cava superior (C), and left subclavian artery (D), were carefully separated from the substernal goiter, and then thyroidectomy was performed.

The patient underwent total thyroidectomy using a transcervical and full sternotomy approach. The anesthesiologist intubated the patient with a bronchoscope. Although tracheal stenosis was observed, intubation was performed smoothly. Later, the tracheal tube was replaced with an NIM™ EMG endotracheal tube so that intraoperative nerve monitoring could be performed. The patient was placed in a supine position with her neck well extended. A cervical skin incision was made, and a median chest midline incision and full sternotomy were performed. First, we identified the bilateral vagal nerves and confirmed the absence of paralysis with the NIM™. As a preparation for the resection of the substernal goiter, the major blood vessels, including the innominate vein, brachiocephalic trunk, superior vena cava, and left subclavian artery, were carefully separated from the substernal goiter, and then thyroidectomy was performed (Figure 3).

**Figure 4:**
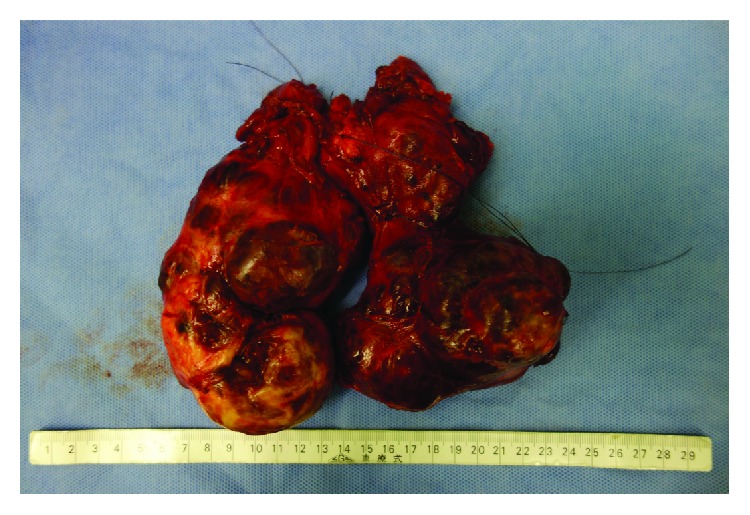
Macroscopic findings. The resected thyroid had multiple nodules. The goiter weighed 614 g, and its dimensions were as follows: width—165 mm, length—160 mm, and thickness—60 mm.

The right superior thyroid pedicle and right middle thyroid vein were ligated and dissected to allow the right thyroid lobe to be rotated to gain a view of the recurrent laryngeal nerve (RLN) from the lateral aspect of the thyroid gland, but the goiter prevented the right thyroid lobe from being rotated. It was difficult to identify the right RLN, so we decided to try to exteriorize the left thyroid lobe, which was smaller than the right thyroid lobe. The left superior thyroid pedicle and the left middle thyroid vein were ligated and dissected. The left thyroid lobe was more mobile than the right thyroid lobe, and the left RLN could be identified by rotating the left thyroid lobe in the medial direction. The NIM™ was effective at identifying the RLN. After identifying the left RLN, the left lower thyroid artery was ligated and dissected. The left RLN was carefully separated from the dorsal side of the left thyroid lobe and the substernal goiter so as not to cause any damage. The substernal goiter, which was connected to the left thyroid lobe, was pulled in the cranial direction, and the part adhering to the surrounding tissue, particularly the tissue between the goiter and the innominate vein, was dissected by ligation and coagulation with an energy device. Subsequently, the left thyroid lobe was also separated from the trachea. The exteriorization of the left thyroid lobe improved the mobility of the right thyroid lobe, and the right RLN was identified by dislocating the right upper pole to the caudal side. We carefully separated the right RLN from the goiter and ligated and dissected the right lower thyroid artery. We pulled the substernal portion of the right thyroid lobe gradually; separated the tissue connected to the goiter, including the left thyroid lobe; and succeeded in moving the substernal goiter in the cranial direction. The remaining attachments between the right thyroid lobe and trachea were broken, and a total thyroidectomy was conducted. We found three parathyroid glands had adhered to the resected thyroid gland, so we performed autotransplantation using the sternocleidomastoid muscle. The wound closed after drains were inserted in the neck and mediastinum. After the surgery, the patient was extubated immediately because no respiratory tract problems (e.g., tracheomalacia) were noted. The total duration of the operation was 9 h and 22 min, and the total amount of intraoperative blood loss was 3298 ml. The resected thyroid weighed 614 g (Figure 4).

Postoperative transient hypoparathyroidism was observed. Routine treatment with calcium (3 g daily orally) and 1 alpha-hydroxyvitamin D3 (2 *μ*g daily orally) was administered. The patient was discharged home on the 9th postoperative day on levothyroxine (100 *μ*g daily orally). A histopathological examination did not reveal any signs of malignancy, and so the lesion was diagnosed as an adenomatous goiter.

## 3. Discussion

In this case, the substernal goiter extended into both sides of the thorax, and both the left and the right parts of the goiter extended deep enough to reach the bifurcation of the trachea and spread widely in the mediastinum. It is very rare to encounter such a large substernal goiter. Surgery was performed via a transcervical and full sternotomy approach without additional incision such as thoracotomy. The goiter had widely adhered to the major blood vessels and had abundant blood flow. Removal of the goiter from the surrounding tissues took a long time and resulted in bleeding in large amounts from the goiter itself and the surrounding tissues, but postoperative complications, such as RLN palsy or injury to major blood vessels, were avoided. We considered that a transcervical and full sternotomy approach was a necessary procedure for this case.

Substernal goiters are found in 5.1–15.7% of patients who undergo thyroid surgery [1, 2, 5–9]. Various definitions of substernal goiter have been reported, for example, goiters in which >50% of the lesion is located in the thorax [3, 4], those in which any part of the lesion extends below the thoracic inlet [1, 2, 5] and those that extend ≥3 cm below the sternal notch or extend below the fourth thoracic vertebra [10, 11]. Estimates of the frequency of substernal goiters vary because of the differences in these definitions. Substernal goiters exhibit a 1.6 times higher frequency in females than in males, and the mean age at diagnosis is reported to be in the 6th decade of life [2, 3]. The vast majority of substernal goiters (85–90%) are located in the anterior mediastinum, with the remainder (10–15%) located in the posterior mediastinum [2, 12]. Lin et al. reported that substernal goiters displaying unilateral extension were more common than those demonstrating bilateral extension [1]. In the current case, the substernal goiter extended into both sides of the thorax. It extended further to the right side than to the left side, possibly because of the anatomical location of the aortic arch. When substernal goiters expand into the inferior mediastinum, less resistance is encountered during extension to the right side of the trachea owing to the relatively loose areolar tissue found in this region. A mediastinal goiter location is most frequently the result of the natural descent of a goiter from a primary cervical site facilitated by negative intrathoracic pressure, gravity, and a large potential mediastinal space [13].

In many cases, the substernal goiter grows slowly, so it remains asymptomatic for many years. Approximately 20–40% of substernal goiters are discovered as an incidental finding on a radiographic examination, such as a chest X-ray. The most common symptoms are related to compression of the airway or esophagus and include dyspnea, choking, an inability to sleep comfortably, dysphagia, and hoarseness [2]. These compressive symptoms are usually indications for surgery. In addition, radiological signs of compression, such as tracheal deviation, are also indications for surgery. In some patients, the correlations between symptoms and the presence/absence of tracheal deviation, the size of the goiter, or the extent of substernal extension as assessed by CT are poor [14]. In our case, the patient did not have any symptoms in spite of severe airway constriction being discovered on CT. Thus, the possibility of airway occlusion should be considered, even if no such findings are noted on radiographic examinations.

CT is very useful for evaluating substernal goiters. Several evaluation systems have been reported [5, 12]. Huins et al. proposed a new 3-grade classification system for substernal goiters based on a systematic review of the complications and management of the condition. Substernal goiters that extend from the aortic arch to the pericardium cannot be approached via a cervical incision alone without risk. Mercante et al. reported a CT-based classification of substernal goiters, which considers the following 3 spatial dimensions: the craniocaudal (sagittal), anteroposterior (axial), and laterolateral (coronal) planes. Statistical analysis confirmed that an extracervical approach represents a major risk in cases of grade 2 or 3 substernal goiters, i.e., those located below the convexity of the aortic arch and, in cases of type C substernal goiters, i.e., those found in a retrotracheal position.

The general consensus is that substernal goiters are best managed surgically. Substernal goiters can be safely treated with thyroidectomy through a cervical incision in almost all cases [9]. The probability that a sternotomy will be needed during thyroidectomy has been reported to range from 0.6 to 9.5% [1, 2, 5, 9], but when endocrine surgeons perform surgery, it is estimated to be around 2% [13]. The predictive factors for sternotomy include involvement of the posterior mediastinum, the extension of the goiter into the aortic arch, recurrent goiters, an ectopic thyroid, superior vena cava obstruction, malignancy with local involvement, and emergent airway obstruction [1, 2]. Although some substernal goiters that extend to the aortic arch can be removed via a transcervical approach, it might not be possible to do so if the diameter of the goiter is >10 cm or significantly greater than that of the thoracic inlet [14]. In the present case, the substernal goiter extended to both sides of the thorax and had a larger diameter than the thoracic inlet, and airway constriction was revealed, so a full sternotomy was required. A full sternotomy provides excellent exposure, can be performed simply and quickly, and is associated with a low morbidity rate [15].

Due to their localization, it is difficult to determine whether substernal goiters are benign or malignant before surgery. Lin et al. reported that the probability of malignancy is assumed to be around 10%, which is not significantly different from that of cervical goiters [1]. In the current case, a histopathological examination did not reveal any signs of malignancy, and so the lesion was diagnosed as an adenomatous goiter. On the other hand, several authors have recommended surgery for asymptomatic patients with substernal goiters because these lesions carry an increased risk of malignancy compared with cervical goiters [8]. In a multicenter study involving 19,662 patients, the frequency of malignancy was significantly higher among goiters that presented with cervicomediastinal extension (22.4%) and was even higher among the patients treated with manubriotomy (36.2%).

As for the postoperative complications of surgery for substernal goiters, they have been reported to include pneumonia, atelectasis, pneumothorax, pleural effusion, and innominate vein injuries, etc., which seem to be characteristic complications of mediastinal surgical procedures [16]. The most common complication is transient hypocalcemia, and its frequency is reported to range from 2 to 28.9%. On the other hand, it was reported that the frequency of permanent hypocalcemia ranges from 0 to 8.1% [1, 2, 5, 6, 16]. In the present case, transient hypocalcemia was observed, but it subsequently resolved. The frequencies of transient RLN palsy and permanent RLN palsy were reported to range from 4.7 to 13.8% and from 0 to 4.7%, respectively [1, 2, 5, 16]. It has been demonstrated that permanent RLN palsy is more common on the right side than on the left side due to the anatomical location of the RLN, and blind manipulation without visual nerve identification probably increases the risk of RLN damage [1]. Raffaelli et al. reported that blind finger luxation of the thyroid lobes should be avoided to reduce the risk of RLN damage and dangerous and difficult-to-control mediastinal hemorrhaging [9]. In the current case, RLN palsy was avoided by carrying out surgery safely via a full sternotomy and using an intraoperative nerve monitor (NIM™) during the thyroidectomy. We consider that the NIM™ is very useful for preventing RLN palsy in cases involving very large goiters like the present case.

The presence of a substernal goiter, especially one that persists for more than 5 years and/or causes significant tracheal compression, is probably a risk factor for tracheomalacia and tracheostomy. Tracheomalacia combined with a substernal goiter is an infrequent condition, and many cases of tracheomalacia can be managed without a tracheostomy [13]. Clear associations were detected between the extent of substernal goiters and the incidence of tracheomalacia or the need for an intrathoracic approach, with a more than a 10-fold increase in the incidence of tracheomalacia/the need for an intrathoracic approach seen in cases in which the substernal goiter extended beyond the aortic arch [12]. Fortunately, tracheomalacia did not occur in this case, and the patient's postoperative respiratory state was stable.

## 4. Conclusions

Most substernal goiters can be managed through a transcervical approach, but a full sternotomy is required when a substernal goiter extending to both sides of the thorax and/or has a larger diameter than the thoracic inlet or when airway constriction is revealed. A full sternotomy provides excellent exposure and can help reduce the risk of complications, such as RLN palsy and injuries to major blood vessels. Intraoperative nerve monitoring can also help reduce the risk of RLN palsy.
